# Novel protein-truncating variants of a chromatin-modifying gene *MSL2* in syndromic neurodevelopmental disorders

**DOI:** 10.1038/s41431-024-01576-0

**Published:** 2024-05-03

**Authors:** Xiaona Lu, Kim Ng, Filippo Pinto e Vairo, James Collins, Ronald Cohn, Kacie Riley, Katherine Agre, Ralitza Gavrilova, Eric W. Klee, Jill A. Rosenfeld, Yong-hui Jiang

**Affiliations:** 1 Department of Genetics, Yale University School of Medicine, New Haven, CT 06520 USA; 2 Pediatric Medical Genetics, Department of Pediatrics, Duke University School of Medicine, Durham, NC USA; 3 Department of Clinical Genomics and Center for Individualized Medicine, Mayo Clinic, 200 First Street SW, Rochester, MN 55905 USA; 4 Mercy Hospital, Pediatric Neurology, St Louis, MO USA; 5 SickKids, The Hospital for Sick Children, 555 University Ave Toronto, Toronto, ON M5G 1X8 Canada; 6 Department of Molecular and Human Genetics, Baylor College of Medicine, Houston, TX 77030 USA; 7 Baylor Genetics Laboratories, Houston, TX 77030 USA; 8 Pediatrics, Yale University School of Medicine, New Haven, CT 06520 USA; 9 Neuroscience, Yale University School of Medicine, New Haven, CT 06520 USA; 10 Professor of Genetics, Neuroscience, & Pediatrics Chief of Medical Genetics, Yale University School of Medicine Yale New Haven Hospital, New Haven, CT 06520 USA

**Keywords:** Genetics, Diseases

## Abstract

Numerous large scale genomic studies have uncovered rare but recurrent pathogenetic variants in a significant number of genes encoding epigenetic machinery in cases with neurodevelopmental disorders (NDD) especially autism spectrum disorder (ASD). These findings provide strong support for the functional importance of epigenetic regulators in neurodevelopment. After the clinical genomics evaluation of the patients using exome sequencing, we have identified, three novel protein-truncating variants (PTVs) in the *MSL2* gene (OMIM: 614802) which encodes a chromatin modifying enzyme. MSL2 modifies chromatin through both mono-ubiquitination of histone 2B on lysine 34 (K34) and acetylation of histone H4 on lysine 16 (K16). We reported first time the detailed clinical features associated with 3 *MSL2* PTVs. There are 15 PTVs (13 de novo) reported from the large genomics studies (12 cases) or ClinVar (3 cases) of NDD, ASD, and developmental disorders (DD) but the specific clinical features for these cases are not described. Taken together, our descriptions of dysmorphic face and other features support the causal role of *MSL2* in a likely syndromic neurodevelopmental disorder and add *MSL2* to a growing list of epigenetic genes implicated in ASD.

## Introduction

Genomics studies of neurodevelopmental disorders (NDD), in particular autism spectrum disorder (ASD), have uncovered rare but recurrent pathogenic  variants in a growing list of causal genes [1–3]. The clinical application of genomic copy number variants (CNVs) exome sequencing (ES), and whole genome sequencing (WGS) are routinely offered to patients with ASD and other NDD [4]. Approximately 15% of patients with ASD are found to have an identifiable genetic cause involving about 200 genes [1–4]. Notably, pathogenic variants in a significant number of genes encoding proteins in epigenetic machinery have been found in patients with ASD and NDD [5–7].

After the evaluation of the patients in clinic, we have identified protein truncating variants (PTVs) of *MSL2* (male-specific lethal 2 in Drosophila) ES in three subjects with ASD and developmental disorders (DD). *MSL2* encodes a protein that is a subunit of a protein complex for dosage compensation for sex chromosome specific genes in Drosophila [8]. MSL2 is implicated in histone modifications through ubiquitination and acetylation [9, 10]. The discovery of predicted loss-of-function variants in *MSL2* supports the pathogenicity of these PTVs for NDD and ASD and an as yet undefined important function of MSL2 in brain development.

## Clinical presentations and genetic findings

Three unrelated cases were evaluated in clinics. Case 1 had clinical trio ES. Patients 2 and 3 had proband-only ES. Clinical information was provided by the referring physicians or extracted from the clinical notes.

### Patient 1

This patient is a 6-year-old girl with ASD, DD, and other behavioral problems including anxiety and self-injurious behavior (Unpublished abstract, Dept. of Pediatrics, Duke University). She was a product of a term pregnancy and normal delivery. Her newborn course was complicated by central hypotonia and feeding difficulties. Family history was significant for learning difficulties in the father and maternal half-brother. Her current weight was 18.9 kg (47th percentile), height was 118.2 cm (17th percentile) and head circumference was 49.6 cm (96th percentile). Overall, her motor development was delayed. Mild facial dysmorphism was noted with the features of down-slanting palpebral fissures, webbed neck, flat midface, low-set ears, micrognathia, down-turned mouth, and webbed neck. The diagnosis of ASD was confirmed with ADOS-2. Her standard score on WPPSI-IV was 75 (5th percentile). The score for Visual Perception was 45 (2nd percentile), for the Motor Coordination was 63 (1st percentile) and Visual Motor Integration was 77 (6th percentile). The score on PPVT-IV test was 80 (9th percentile). The performance on Visuomotor Precision Time was average (score = 3), but on the Visuomotor Precision Combined, it was extremely poor (score = 3). No history of seizure. MRI of brain was unremarkable. The comparison of clinical features for 3 patients was summarized in Table 1.

**Table 1:** Summary of clinical features associated with *MSL2* PTVs.

	Patient 1	Patient 2	Patient 3
**Age (year)**	6	11	16
**Sex**	female	male	female
***MSL2*** **variants**	(NM_018133.3):c.796-797delCT, p.(Leu266Valfs*5); De novo	(NM_018133.4):c.1047-1050del, p.Ser349Argfs*23; mother is negative and father not tested	(NM_018133.4):c.67 G > T, p.Gly23*; parents were not tested
**Growth**	normal	normal	normal
**Hypotonia**	Yes	Yes	Yes
**Face dysmorphism**	Yes	Yes	No
**Other physical features**	Webbed neck	pectus excavatum deformity	joint hypermobility
**DD**	global developmental delay	global developmental delay	Motor delay
**ID**	Yes	Yes	No
**ASD**	Yes	not evaluated	No
**Other behavioral disorders**	anxiety and self-injurious behavior	self-injurious and aggressive behavior	regression of motor skills
**Seizure**	No	Yes	No
**EEG**	not evaluated	abnormal	not evaluated
**MRI of brain**	unremarkable	abnormal	not evaluated
**Family history**	negative	negative	similar symptom in mother and maternal grand mother

**Fig. 1:**
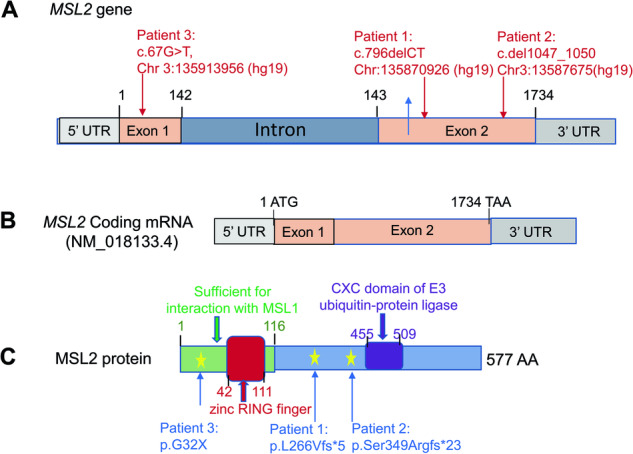
Schematic illustrating *MSL2* gene, mRNA, and protien structure and as well as annotatoins of pathogeneic variants. **A** *MSL2* gene structure and postions of pathogentic variants. **B** mRNA of *MSL2* (Noted: NM_018133.3 was used to annotate the variant of patient #1 and NM_018133.4 was used for the patient #2 and #3). **C** MSL2 protein structure and pathogenic variants.

Patient had negative *FMR1* and chromosome microarray (CMA). Trio ES identified a de novo indel of c.796-797delCT in the *MSL2* gene (NM_018133.3) (Fig. 1A, B). The open reading frame (ORF) analysis predicted that mutation results in a frameshift and truncated protein (p.Leu266Valfs*5). The truncated protein, if stable, would miss the C-terminal CXC domain of E3 ubiquitin-protein ligase of MSL2 protein (Fig. 1C).

### Patient 2

**Fig. 2:**
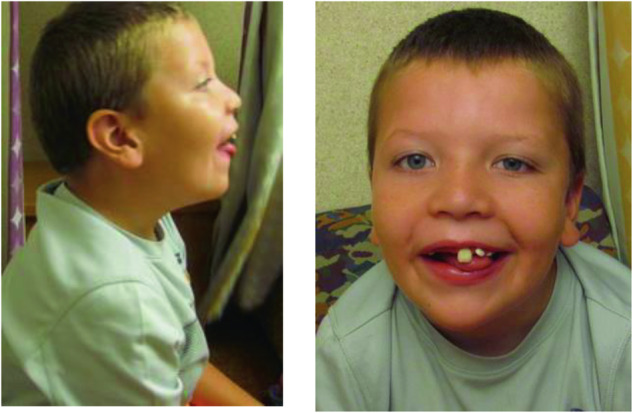
Front and side facial profile of patient 2 showing slightly down-slanting palpebral fissures, flat midface, mild low-set ears, wide and smooth philtrum, and micrognathia.

The patient is an 11-year-old boy referred for the evaluation of global developmental delay, intractable seizure, abnormal brain MRI, and behavioral abnormalities. The pregnancy was complicated by an abnormal prenatal ultrasound showing brain abnormalities. He was born at full term via repeat C-section. Growth parameters were normal. He walked around 15 months of age and developed words between 2 and 3 years of age. He has self-injurious and aggressive behavior. Seizure started at 18 months of age and was not fully controlled with the seizure medications. MRI of brain showed cystic changes in the right subcortical and periventricular white matter. He also has left hemispheric atrophy with ex vacuo dilatation of the left lateral ventricle, and cerebellar atrophy. EEG was abnormal with dysrhythmia and bitemporal discharges. Mild dysmorphisms include hypertelorism, epicanthal folds, slightly down-slanting palpebral fissures, flat midface, mild low-set ears, wide and smooth philtrum, and micrognathia (Fig. 2). The pectus excavatum were also noted (Table 1).

The patient had negative CMA, *FMR1*, and biochemical tests. ES identified a 4 bp deletion of c.1047_1050delTGAG in the *MSL2* gene (NM_018133.4) (Fig. 1A, B). The ORF analysis predicted that variant results in a truncated protein of p.Ser349Argfs*23 (Fig. 1C). The truncated protein, if stable, is predicted to miss the CXC domain of E3 ubiquitin-protein ligase (Fig. 1C). Parental test of mother was negative but father was not available for the test.

### Patient 3

This patient is a 16-year-old female with central hypotonia, exercise intolerance, and difficulty swallowing. She was born at full term via cesarean section with no perinatal complications. She did not have facial dysmorphism. She had feeding issues and required soft or pureed diet. She walked at age 16 months and motor development was delayed. Central hypotonia was first noted at 18 months of age and she has since regressed her motor milestones. Growth was normal. She had normal language development. No significant cognitive impairment and abnormal behaviors were reported. She was ambulatory with significant weakness at teenage that required wheelchair assistance. She had hyper-extensibility of joints. Her muscle CPK was normal. Electromyography, nerve conduction study, ECHO, and EKG were negative. Her polysomnogram was normal. She had mild thoracic scoliosis ( < 10 degrees). There was a family history of similar symptoms in the mother and maternal grandmother. Muscle biopsy in mother was reported as negative.

ES revealed a heterozygous novel nonsense variant in exon 1 (c.67 G > T, p.Gly23*) of the *MSL2* gene (NM_018133.4) (Fig. 1A, B). The open reading frame (ORF) analysis predicted that mutant protein miss all known functional domains for MSL2 protein (Fig. 1C). Parental test for the variant was not completed due to family relocation.

## The summary of PTVs of *MSL2* from other genomic studies of ASD, NDD, and DD

We found a total of 12 de novo PTVs of *MSL2* from 3 large scale of genomics studies (Table 2). In the study by Kaplanis et al. [11], 45,221 coding and splicing de novo mutations (DNMs) in 31,058 individuals were pooled from 3 cohorts: GeneDx (GDX), the Deciphering Developmental Disorders (DDD) study and Radboud University Medical Center (RUMC). Nine de novo PTVs and 3 missense variants of *MSL2* were described from these cohorts with DD. In study by Zhang et al. [12], a targeted sequencing of 547 genes was performed for 1102 subjects with NDD. Two de novo PTVs of *MSL2* were identified. In the study by Iossifov et al. [13], ES of 2508 subjects with ASD was performed. One PTV of *MSL2* was identified. Unfortunately, the specific clinical features other than DD/NDD/ASD were not described in these studies. There are 3 PTVs (2 de novo) deposited in ClinVar (Table 2). The indication for genetic testing is ASD for 2 cases and global developmental delay for other case. Collectively, the report of these *MSL2* PTVs provide additional evidence supporting the causal role of *MSL2* in ASD/NDD.

**Table 2:** Summary of *MSL2* PTVs from other genomics studies of ASD, NDD, and DD and ClinVar.

Cohort	Chr.	Gene	Position(hg19)	NM_018133.4	Sequence change	Consequence		Disease
**Kanlanis et al. (2020)**								
GDX	Chr.3	*MSL2*	130871025	exon 2	de novo TCAGA > T		Frameshift	DD
	Chr.3	*MSL2*	135871051	exon 2	de novo A > AACAGTATT		Frameshift	DD
	Chr.3	*MSL2*	135871109	exon 2	de novo AATCT > A		Frameshift	DD
	Chr.3	*MSL2*	135871014	exon 2	de Novo GTGGCAGGCTGTCAGACAGA > G		Frameshift	DD
	Chr.3	*MSL2*	135870925	exon 2	de novo CAG > C		Frameshift	DD
	Chr.3	*MSL2*	135871072	exon 2	de novo CGT > C		Frameshift	DD
DDD1	Chr.3	*MSL2*	135870925	exon 2	de novo CAGAGAG > CAGAG		Frameshift	DD
	Chr.3	*MSL2*	135871015	exon 2	de novo TGGCAGGC > TGGCAGGCAGGC		Frameshift	DD
RUMC	Chr.3	*MSL2*	135870956	exon 2	de novo del A		Frameshift	DD
**Zhang et al**. [12]								
	Chr.3 Chr.3	*MSL2* *MSL2*	135870926	exon 2 exon 2	de novo del AG		Frameshift	NDD
			135870044		de novo del G		Frameshift	NDD
**Iossifov et al**. [13]								
	Chr.3	*MSL2*	135871026	exon 2	de novo TCAGA > T		Frameshift	ASD
**ClinVar**								
2265934	Chr.3	*MSL2*	135871201	exon 2	unknown dup T		Frameshift	NDD
638594	Chr.3	*MSL2*	135871026	exon 2	Unknown del CAGA		Frameshift	ASD
2499549	Chr.3	*MSL2*	135870621	exon 2	de novo del C		Frameshift	ASD

*DD* developmental delay, *NDD* neurodevelopmental disorders, *ASD* autism spectrum disorder, *GDX* GeneDx, *DDD* the Deciphering Developmental Disorders study, *RUMC* Radboud University Medical Center, Chr. Chromosome.

## Discussion

We presented, for the first time, detailed clinical presentations of 3 patients with PTVs of *MSL2*. We also identified three PTVs in ClinVar that are classified as variant of uncertain significance (VUS). The indication of genetic testing for two cases is ASD and other is DD. Together with 12 de novo PTVs of *MSL2* curated from 3 large cohorts of clinical genetic testing or research studies, our finding provides strong support for a pathogenic role PTVs of *MSL2* in NDD and ASD. The detail clinical features associated with PTVs of *MSL2* are not available in other cases. Because of the report of dysmorphic facial features in our cohort, pathogenic variant in *MSL2* may represent a syndromic NDD. Similar to many recent discovered genes implicated in NDD and ASD, the clinical features associated with PTVs of *MSL2* are variable. For example, no apparent cognitive impairment was observed patient 3 with the caveat that the patient is lost for follow up. This is somewhat unexpected because nonsense variant at very beginning of coding exon in patient 3 is predicted to result in complete loss of function of MSL2 protein. Further neurodevelopmental evaluation or nature history are warranted. In a recent report, the protein truncating variants of *MSL2* are characterized as nonsense-mediated decay escaping variants (ASHG abstract#335 2022). These findings suggest that PTVs of *MSL2* do not necessarily result in a loss of function mechanism as predicted. The possibility that the truncating variant at C-terminus result in a gain of function mechanism could be considered because of the presence of a stable and truncated MSL2 protein with zinc finger domain in the N-terminus. The similar mechanism has been described for *PPM1D* truncating mutations associated NDD [14]. The genotype phenotype correlation in cases with *MSL2* PTVs may be more complex and required more functional studies.

*MSL2* was first identified in Drosophila, designated male-specific lethal 2. *MSL2* is only expressed in male Drosophila and encodes a protein that functions as part of a multi-subunit dosage compensation complex on the X chromosome [8, 15]. The function of human MSL2 remains poorly characterized. In mammalian cells, MSL2 is a subunit of a protein complex including MSL1, MSL2, and MSL3 that implicates in chromatin modifications [16, 17]. MSL2 is implicated both in acetylation of histone H4 on lys16 (K16) [9] and in ubiquitination of histone H2B on K34 (H2Bk34) [10]. H2BK34 ubiquitination increased H2BK120 ubiquitination indirectly by facilitating chromatin association of both RNF20 and RNF40 [9, 18]. To our knowledge, this is the first example that two different types of histone modifications mediated by a single protein and implicated in NDD. Recent studies of *MSL3*, a close family gene of *MSL2*, have shed some light into the importance of the MSL complex in neurodevelopment. Pathogenic mutations of *MSL3* have been reported in two cohorts with Basilicata-Akhtar syndrome [19, 20]. Majority of variants in *MSL3* are de novo but rare inherited variants are also observed. The reduction of histone acetylation of H4K16 is seen in skin fibroblasts of these patients [19]. The clinical features associated with *MSL2* and *MSL3* pathogenic variants overlap. These include the developmental delay, intellectual disability, ASD, hypotonia, epicanthal folds, low set of ears, pectus excavatum and MRI brain finding of dilation of ventricle. Further functional studies of variants in *MSL2* patients are necessary to investigate the perturbations induced by these variants in *MSL2*.

## Data Availability

The sequence variants for 3 subjects have been submitted to ClinVar. The submission ID is SUB13875470 for case 1, SUB13862870 for case 2, and SUB13861384 for case 3 (https://www.ncbi.nlm.nih.gov/clinvar/).

## References

[1] Zhou X, Feliciano P, Shu C, Wang T, Astrovskaya I, Hall JB (2022). Integrating de novo and inherited variants in 42,607 autism cases identifies mutations in new moderate-risk genes. Nat Genet.

[2] Fu JM, Satterstrom FK, Peng M, Brand H, Collins RL, Dong S (2022). Rare coding variation provides insight into the genetic architecture and phenotypic context of autism. Nat Genet.

[3] Satterstrom FK, Kosmicki JA, Wang J, Breen MS, De Rubeis S, An JY (2020). Large-scale exome sequencing study implicates both developmental and functional changes in the neurobiology of autism. Cell.

[4] Jiang YH, Wang Y, Xiu X, Choy KW, Pursley AN, Cheung SW (2014). Genetic diagnosis of autism spectrum disorders: the opportunity and challenge in the genomics era. Crit Rev Clin Lab Sci.

[5] Duffney LJ, Valdez P, Tremblay MW, Cao X, Montgomery S, McConkie-Rosell A (2018). Epigenetics and autism spectrum disorder: a report of an autism case with mutation in H1 linker histone HIST1H1E and literature review. Am J Med Genet B Neuropsychiatr Genet.

[6] Tremblay MW, Jiang YH (2019). DNA methylation and susceptibility to autism spectrum disorder. Annu Rev Med.

[7] Grayson DR, Guidotti A (2016). Merging data from genetic and epigenetic approaches to better understand autistic spectrum disorder. Epigenomics.

[8] Belote JM (1983). Male-specific lethal mutations of DROSOPHILA MELANOGASTER. II. Parameters of gene action during male development. Genetics.

[9] Smith ER, Cayrou C, Huang R, Lane WS, Cote J, Lucchesi JC (2005). A human protein complex homologous to the Drosophila MSL complex is responsible for the majority of histone H4 acetylation at lysine 16. Mol Cell Biol.

[10] Wu L, Zee BM, Wang Y, Garcia BA, Dou Y (2011). The RING finger protein MSL2 in the MOF complex is an E3 ubiquitin ligase for H2B K34 and is involved in crosstalk with H3 K4 and K79 methylation. Mol Cell.

[11] Kaplanis J, Samocha KE, Wiel L, Zhang Z, Arvai KJ, Eberhardt RY (2020). Evidence for 28 genetic disorders discovered by combining healthcare and research data. Nature.

[12] Zhang Y, Wang T, Wang Y, Xia K, Li J, Sun Z (2021). Targeted sequencing and integrative analysis to prioritize candidate genes in neurodevelopmental disorders. Mol Neurobiol.

[13] Iossifov I, O’Roak BJ, Sanders SJ, Ronemus M, Krumm N, Levy D (2014). The contribution of de novo coding mutations to autism spectrum disorder. Nature.

[14] Kleiblova P, Shaltiel IA, Benada J, Ševčík J, Pecháčková S, Pohlreich P (2013). Gain-of-function mutations of PPM1D/Wip1 impair the p53-dependent G1 checkpoint. J Cell Biol.

[15] Belote JM, Lucchesi JC (1980). Male-specific lethal mutations of Drosophila melanogaster. Genetics.

[16] Valsecchi CIK, Basilicata MF, Semplicio G, Georgiev P, Gutierrez NM, Akhtar A (2018). Facultative dosage compensation of developmental genes on autosomes in Drosophila and mouse embryonic stem cells. Nat Commun.

[17] Tikhonova E, Mariasina S, Efimov S, Polshakov V, Maksimenko O, Georgiev P (2022). Structural basis for interaction between CLAMP and MSL2 proteins involved in the specific recruitment of the dosage compensation complex in Drosophila. Nucleic Acids Res.

[18] Straub T, Gilfillan GD, Maier VK, Becker PB (2005). The Drosophila MSL complex activates the transcription of target genes. Genes Dev.

[19] Basilicata MF, Bruel AL, Semplicio G, Valsecchi CIK, Aktaş T, Duffourd Y (2018). De novo mutations in MSL3 cause an X-linked syndrome marked by impaired histone H4 lysine 16 acetylation. Nat Genet.

[20] Brunet T, McWalter K, Mayerhanser K, Anbouba GM, Armstrong-Javors A, Bader I (2021). Defining the genotypic and phenotypic spectrum of X-linked MSL3-related disorder. Genet Med.

